# Quantifying the economic burden of malaria in Nigeria using the willingness to pay approach

**DOI:** 10.1186/1478-7547-5-6

**Published:** 2007-05-22

**Authors:** Ayodele Jimoh, Oluyemi Sofola, Amos Petu, Tuoyo Okorosobo

**Affiliations:** 1 Department of Economics, University of Ilorin, Ilorin, Nigeria; 2 National Malaria Control Programme, Federal Ministry of Health, Abuja, Nigeria; 3 World Health Organization, Abuja, Nigeria; 4 Regional Office for Africa, World Health Organization, Harare, Zimbabwe

## Abstract

**Background:**

Malaria illness imposes great burden on the society as it has adverse effects on the physical, mental and social well being of the people as well as on the economic development of the nation.

**Methods:**

The study uses the Willingness To Pay (WTP) approach to evaluate the burden of malaria in Nigeria.

**Results:**

The results indicate that households would be prepared to pay an average of about Naira 1,112 (USD 9.3) per month for the treatment of malaria. This is about Naira 427 (USD 3.6) in excess of the average expenditure they currently make on malaria treatment per month. Similarly, households are willing to pay on the average a sum of Naira 7,324 (USD 61) per month for the control of malaria. Again, this is an excess of about Naira 2,715 (USD 22.6) over the cost they currently bear (protection, treatment and indirect costs), and it represents households' average valuation of their intangible costs of malaria illness. This amount represents about Naira 611.7 (USD 5.1) per head per month and Naira 7,340 (USD 61.2) per year. For a country with a population of about 120 million this translates to about Naira 880,801 million per annum representing about 12.0 per cent of Gross Domestic Product. Hence, the malaria burden in Nigeria is enormous and has a devastating impact on economic growth.

**Conclusion:**

In the long term, it is important to recognize that health and poverty are closely linked. Reducing the burden of malaria in Nigeria will help to contribute to the economic well-being of communities; and poverty-reduction will be an essential input into improving health. National malaria control programme in Nigeria and their partners need to recognize these links, and identify mechanisms for ensuring that the poorest have access to essential health interventions.

## Background

Malaria disease is caused by parasites that are spread by mosquitoes. The anopheles mosquitoes transmit the malaria parasites that cause malaria in humans. In adults, its common symptoms are headaches, weakness, fever, aches and pains, high body temperature (chills and rigors) and bitterness of the mouth (and loss of appetite) while in children, in addition to the above-mentioned symptoms, it may also manifests in more than normal sleeping, nausea and vomiting. It is a serious disease affecting children and adults but it consequences are graver among children and pregnant women.

Nigeria is known for high prevalence of malaria [1-3] and it is a leading cause of morbidity and mortality in the country [2]. Available records show that at least 50 per cent of the population of Nigeria suffers from at least one episode of malaria each year and malaria accounts for over 45 per cent of all out-patient visits[2,4]. It is reported that malaria prevalence (notified cases) in 2000 was about 2.4 million [2]. The disease accounts for 25 per cent of infant mortality and 30 per cent of childhood mortality in Nigeria [2,4]. Therefore, it imposes great burden on the country in terms of pains and trauma suffered by its victims as well as loss in outputs and cost of treatments [5].

The disease is often treated in Nigeria by self-medication, the use local herbs, use of the services of spiritualists/traditional priests or/and the use of clinic/hospital services. Similarly, common prevention measures include use of medicine (prophylaxis), insecticides (coils and sprays), ordinary mosquito nets, insecticide-treated nets (ITNs) and widow and door nets.

Nigeria can be divided to three major malaria epidemiological zones, namely, forest, savannah and grass-land zones. The forest zone consists of coastal areas stretching from Lagos in the South-Western Nigeria to the forest areas in the Eastern Nigeria up to the Northern portion of the forest zone of Oyo state. The Savannah zone consists of areas north of Oyo state to the central areas of Kogi and Benue states and the Grass-land zones consists of the most northern parts of Nigeria – Katsina state and areas to its north.

The most dominant species of anopheles mosquito in Nigeria are *anopheles funestus*, *anopheles gambiae complex, anopheles arabiensis *and *anopheles melas*. The dominant vector in the forest zone is *anopheles melas *while the dominant vectors in the savannah zone are a combination of *anopheles melas *and *anopheles arsbiensis; *the dominant vector in the grass-land zone is *anopheles arsbiensis*. Though *Plasmodium falciparum *is reported in all zones, it is not rampant.

There have been some global responses to the devastating effects of malaria. These include the establishment of the Roll Back Malaria partnership by the World Health Organization (WHO) and the Global Fund to fight AIDS, Tuberculosis and Malaria (GFATM). Domestically, the government of Nigeria has subscribed to some known malaria control and prevention measures, including the free distribution of ITNs to the vulnerable groups. A major policy issue is how to put in place a programme of malaria treatment, control and prevention that is fiscally sustainable [5]. Resolving such a policy issue will be facilitated if the malaria burden is quantified and public willingness to pay for the respective components of malaria programmes are known but such evidence have been scanty [3].

It is, therefore, clear that while is now generally accepted that malaria is a serious problem in Africa in general and in Nigeria in particular [1-4], evidence on the magnitude of the malaria burden in Nigeria is scanty and their value for generalization limited because of their limited scope [3]. Besides, there is currently no measure of the intangible burden of malaria in Nigeria.

One of the approaches to measuring the burden of a disease is the Willingness To Pay (WTP) approach. The WTP approach is one of the two subsets of the method of Contingent Valuation (CV). The WTP and its twin concept, the Willingness To Accept (WTA), are the two approaches that are often used to implement the method of contingent valuation of health-care programmes [9,10]. The method of CV is founded in welfare economics and in value theory in particular.

It has been suggested that CV is a method of choice when valuing health programmes for the purposes of decision making and priority setting in the health-care sector [11,12]. It has been used widely to value public safety, disease prevention and control programmes (or services in general), and to value health outcomes or states [13-15].

The CV method in general and the WTP in particular, is particularly suitable for evaluating the burden (or cost) of malaria and especially for valuing malaria control programme. However, because WTP involves asking individuals to state the maximum amount that they would be willing to pay to acquire a service (or to prevent an undesirable health outcome), it is important that relevant questions be asked in a correct manner and after making available to the respondents all information relevant to making a sound decision; the sample must also be representative.

One advantage that can be derived from using the WTP to value the disease burden of malaria is that it is capable of measuring the intangible costs that neither the production function nor cost of illness approach is equipped to measure [16]. This is because after a respondent knows what it would cost him to treat an episode of malaria and the indirect cost (in terms of lost outputs during the sick days), whatever he states in excess of the sum would reflect his valuation of the pains/trauma, etc. (the intangible costs) that are not contained in the direct and indirect costs. Thus, it is a powerful tool for analysts in providing evidence-based policy prescriptions.

Although some studies have applied the WTP approach to some malaria interventions in Nigeria [17-20], no study to the best of our knowledge has applied WTP to measure intangible burden of malaria nor have there been studies with a wide geographical coverage of Nigeria to allow for reasonable level of generalization. The objective of this study is to fill this gap in our knowledge.

To realize this objective, the rest of this report is structured as follows: section 2 describes the methods used and provides the model specification; section 3 gives the empirical results; and concluding remarks are made in section 4.

## Methods

The sample unit for this study was households. The selected households were asked questions on their demographic characteristics; on how much they spend in protecting themselves against malaria attacks; how much they spend in treating a single malaria episode; and their choice of health-care provider; among others. The responses of the respondents were collected via a structured pre-tested questionnaire which was administered by a set of trained enumerators during an interview session with each household.

In selecting the households, the country was demarcated into her six geo-political zones, with a state selected from each to represent the major malaria zones, namely, Lagos State (the equatorial forest zones), Kwara and Kogi States (Savanna zone), Katsina State (Grass lands) and two eastern states (Eastern forest zones). However, because the two Eastern states selected are in the forest zone which could be adequately represented by Lagos State – which is also in the forest zone – for economy of resource use, the two Eastern States were dropped. Four hundred (400) households in each of the selected 4 states were then selected for the administration of the questionnaires. Consequently, a total of 1,600 questionnaires were to be administered by the enumerators. Of these, eighteen questionnaires were unusable because they are not fully or improperly completed, leaving a total of 1582 questionnaires for analysis.

Each State was partitioned to urban and rural areas to arrive at cities, towns and villages to be selected for the study. At the city/town/village level, the supervisor partitioned each to clusters of low-density and high-density areas. Households within each selected city/town/village cluster were finally selected randomly. The questionnaires were administered in August 2003. A tentatively selected household is first screened to determine whether it had at least a malaria case within one month of the interview. A confirmation of a malaria case is through the respondent's description of the major symptoms experienced by the victim and through the verification of available documents, e.g. prescription forms, laboratory reports, payment receipts, etc. When a malaria case is confirmed to have occurred within a month – a period short enough to avoid recall bias – the interview is continued, otherwise, the interview is terminated and the next house selected.

The study asked questions that are typically asked in cost of illness studies that use cost-of-illness approach [21-23] to these were added the WTP questions. The elicitation format for WTP questions was binary-with-follow-up (BWFU) questions (i.e. a bidding process with yes-or-no options). Respondents were first informed of the malaria prevalence rate in the Nigerian society and those that are at the greatest risk as well as the short-term and long-term effects on them. They were further informed of the cost of treating a malaria attack, and going by their own accounts in their responses to earlier questions, they were reminded of their own current expenditures on treatment and prevention, lost work time, as well as of the usual pains and sufferings that are associated with malaria attacks. Thereafter, they were asked to state the amount they are willing to pay per month for an effective treatment whenever any member of the household had a malaria episode, and what their households are willing to pay for the control of malaria, among other questions [for details of these questions, see Additional file 1].

The responses of the respondents are then analyzed using central measures of tendency (specifically, the mean) to determine the value the Nigerian households attach to different malaria prevention methods, malaria treatments and total malaria control.

The excess of the amount people are willing to pay to malaria eradication and control over what it currently costs to treat and prevent it, would be taken as the household valuation of the intangible costs of malaria illness. Furthermore, it is desirable to investigate what determines the amount that households are willing to pay for the eradication of malaria. This is done using a regression analysis. The details of the regression model used are spelt out below.

### Model specification

What people are willing to pay for the treatment or eradication of malaria is best conceptualized within the framework of the traditional consumption theory. Consequently, the formulation of the model for this study shall proceed accordingly. In this study, we expect that household with high income will be willing to pay relatively more for the treatment and control of malaria. Similarly, households with high level of education should be more aware of the benefits of malaria control and therefore willing to pay more as well as those who assess themselves as relatively well off in relation to others in the community. The basis for including a self assessment variable as an explanatory variable in the model can be justified on two grounds, namely, permanent income and life cycle hypotheses [24,25]. On the basis of both permanent income and life cycle hypotheses, wealth level of a consumer is an important explanation of his consumption expenditures with a higher stock of wealth resulting in higher level of real consumption expenditure from a given level of current real income [24,25]. But experience has shown that most Nigerian respondents would not readily give information on the stock levels of their wealth and a proxy measure will have to be used. It is for the same reason that respondents' incomes are determined via the indirect expenditure approach. In such a circumstance, there may be no better measure of one's stock of wealth than his/her self-assessment. Thus, we expect those who assess themselves to be relatively well-to-do in their societies to be willing to pay more for malaria eradication. We also expect those who currently spend high sum on malaria protection and malaria treatment to be willing to pay more for malaria eradication. Furthermore, we expect households with married persons to be willing to pay more – because pregnant women and children are at the greatest of risk and such households have more of these than households with single persons.

We expect that those using public health facilities, where the cost is relatively cheaper and who sometimes enjoy some subsidies, should be willing to pay relatively less. Also, those who bear high indirect costs (measured either by distances to medical facility, lost work days or number of sick days), should be willing to pay more. And finally, we expect strangers in the community (say, those who have stayed less than one year) to have lower altruistic attachment to the community and therefore less willing to pay for more social-oriented programme components of total malaria control menu but relatively more willing to pay for more self-centered prevention methods like bed nets, window/door nets, etc. Similar specifications are found in the literature. For instance, a model of willingness to pay for insecticide-treated nets (ITNs) in Nigeria included education level, marital status and expenditure to treat malaria as explanatory variables, among others [17]. We note however that the variable "number of household residents" included in the model is highly correlated with other household-size-related variables like household's cost of treating malaria, their cost of protection and the indirect cost of malaria. Its inclusion as an explanatory variable in any model that has the above-mentioned variables as explanatory variables will result in a serious multi-colinearity problem, hence, it was excluded from our model. Similarly, because the income variable is included in our model, it will be superfluous to include annual expenditure on school fees as done in the earlier model. In addition, the importance of wealth level in the willingness to pay specifications is recognized in the literature [5]; we only differ on how best the wealth variable should be measured. We use our measure of wealth because in the settings of the study some households have substantial financial assets that are only known to them while others do not hold financial assets but hold physical assets instead. Consequently, self-assessments by households themselves are expected to give a more representative indicator of their stock of wealth.

Informed by these considerations, we specify household's WTP for malaria control (WTPMC) as a positive function of household's income (Y), its level of education (EDUC), its current cost of malaria protection methods (MPROTEC), its current cost of treating malaria cases (MALCOST), the indirect costs of malaria attacks (INDIRECTCOST) and level self assessment (SELF). Furthermore, WTPMC is specified to be positively related to marital status (MARRIED), negatively related to stranger variable (STRANGER) and public medical facility variable (PUBMED). Thus, we write:

WTPMC = WTPMC (Y, EDUC, MPROTEC, MALCOST, INDIRECTCOST, SELF, MARRIED, STRANGER, PUBMED)..... (1)

where the partial derivatives of the dependent variable with respect to Y, EDUC, IMPROTEC, MALCOST, INDIRECTCOST, and MARRIED are expected to be positive and negative for others (i.e. for SELF, STRANGER and PUBMED). It is worth noting that it is for the same reasons that MALCOST variable enters the model that INDIRECTCOST and PUBMED enter the model – those currently bearing higher cost of the malaria disease are willing to pay more for its eradication or treatment.

### Method of estimation and data measurements

The methods of estimation involve first using the methods of ordinary least squares to obtain initial estimates of parameters. Thereafter, ordinal regression (that uses a multivariate ordered logit procedure) is used to confirm whether or not the regression analysis is appropriate. The results of such analysis indicate the Ordinary Least Square (OLS) regression estimates have the same signs as estimates from ordinal regression. Consequently, the OLS estimates are adopted and reported in this study. Also, the functional form of the model is empirically determined – whether linear or log linear. It is worth noting that taking the log of SELF variable poses no danger as its values varies from 1 to 5. Though a discrete variable, the logs of 1 through 5 are order-preserving and therefore constitute no problem for estimation. However, care and caution should be exercised when interpreting the coefficient of its log value.

Data on variables were measured based on variable description and variable type [see Additional file 2]. However, we provide here further clarifications on some of the variables. First, MARRIED variable takes the value of one for all married households and zero for others. Similarly, STRANGER variable takes the value of one of all households whose length of stay in the community is one year and below and for others zero. The PUBMED variable takes the value of one for households using public medical facilities for the treatment of their malaria cases and zero for all others.

The SELF variable uses codes from 1 to 5, with 1 representing the rich and 5 the very poor, i.e. the higher value means lower status self-assessment. Therefore, a negative co-efficient for SELF variable implies a positive relationship between WTPMC and SELF variable.

The MPROTEC variable was proxied by the household's current cost of spraying the rooms regularly using aerosol insecticide sprays. This is because, although the leading method of protection among the households is using window/door nets (36.3%) {followed by spraying the rooms (28.2%)}, window/door netting is more of a capital expenditure, with a life span of between 2 and 4 years. Spraying of rooms on the other hand is carried out daily in most households using this method. Besides, the average cost of regularly spraying the rooms is the highest when compared with any other specifically mentioned methods. Consequently, the cost of spraying the rooms, being a recurrent expenditure, is expected to have a significant influence on what households are willing to pay for malaria control. Finally, the income variable is measured by the sum of household expenditures and savings because respondents would be reluctant to indicate their income. Using the expenditure approach, detailed questions were asked to determine the income of each household. [see Additional file 3]

## Results

Below, we present the empirical results. First, the results in respect of the average sum that households are willing to pay for major methods of protection against malaria attacks as well as for total eradication of malaria are presented followed by the estimated parameters for equation (1) above.

### Average willingness to pay

Table 1 indicates that households are willing to pay, an average of Naira 1,112 per month (USD 9.3) for the treatment of adult malaria victim and a slightly higher figure of Naira 1,132 (USD 9.4) for a child victim. Furthermore, it shows that they are willing to pay an average Naira1,325 (USD 11) for the supply of bed nets and about Naira 1,068 (USD 8.9) for area spraying. Similarly, it shows that the average sum that households are willing to pay for total eradication of malaria is an average of Naira 7,324 (USD 61).

**Table 1:** Summary statistics of amounts that households are willing to pay

	**N**	**Sum**	**Mean**	**Std. deviation**
Willingness to pay for malaria treatment (Medcare) ADULT	1510	1679300	1112.1192	921.5009
Willingness to pay for malaria treatment (medcare) CHILD	1383	1565100	1131.6703	916.1412
Willingness to pay to avoid (protection) malaria attack (BED NETS)	1412	1870850	1324.9646	1103.5628
Willingness to pay for protection (area spraying, twice a year)	1437	1534750	1068.0237	1084.9464
Willingness to pay for total eradication of malaria	1440	1.1E + 07	7324.1667	9401.0222
Valid N (listwise)	1257			

**Source**: computed in the study

To bring out the message in Table 1 more clearly, the average values therein are put side by side with relevant average actual expenditures made by households in Table 2. The implication of the difference between the actual cost of protection, treatment and indirect cost to the households and what the households are willing to pay on the average for the eradication of malaria represents the household valuation of the intangible costs. This is about Naira 2,715 (USD 22.6) per month per household.

**Table 2:** Estimates of what households are willing to pay and their corresponding actual expenditure

**Cost Items (N)**	**Actual expenditures (N)**	**Amount they are willing to pay (N)**	**excess over actual (N)**
Treatment – Adult^1,2^	685.19	1'112.10	426.91
Treatment – Child	**-**	1'131.70	-
Bed nets	1'000.90	1'325.00	324.10
Window/Door nets	643.86	-	
Area spraying (2/year)	-	1'068.00	
Room spraying	806.89	-	
Eradication of malaria^3^	4'609.19	7'324.20	2'715.01

Notes:

1. Actual is the weighted average cost of obtaining treatment and cure from the three major health-care providers multiplied by average malaria cases per household (1.08). It is calculated that 0.53, 0.07 and 0.40 of patients are treated and cured by self-medication, Herbalist/Spiritualist and Clinic/Hospital respectively.

2. Treatment cost in Clinic/Hospital not involving admission is used.

3. Actual is the sum of protection expenditures, weighted treatment costs and indirect costs – all per household and per month.

**Source: **computed in this study

Similarly, the difference of Naira 427 (USD 3.6) per month in respect of malaria treatment should be interpreted as the extra amount that they are prepared to pay if an organisation is offering malaria treatment insurance policy upon which they could draw as the need arises. An analogous interpretation should be given to the difference of Naira 324 (USD 2.7) per month for bed nets – payment for some inconvenience involved in self procurement of bed nets relative to it being provided.

### Regression results

Tables 3 and 4 present the regression results. Variables that are not present in the estimated equations reported in the Tables are excluded because of their extreme insignificance for efficiency gain in line with standard econometric practice. A comparison of these two tables indicates the log-linear functional form performs better though the two results are fairly good especially for a cross-sectional study. Consequently, our analysis shall be based on results in Table 4. In particular, because the model reported in Table 4 has good statistical properties with estimated parameters all having correct a priori signs – good R^2 ^(0.987), good DW (1.522) and F = 15341.9 – valid inferences could be made from it.

**Table 3:** Estimates of the linear regression model

	**Unstandardized coefficients**		**Coefficients Standardized coefficients**		
			
**Model**	**B**	**Std. error**	**Beta**	**T**	**Sig.**
**1 **Public medical facilities (PUBMED)	-2344.933	581.479	-.130	-4.033	.000
Stranger (STRANGER)	2931.634	1642.068	.033	1.785	.074
Married household (MARRIED)	1754.771	1642.068	.140	3.132	.002
Household highest level of education (EDUC)	482.817	41.124	.576	11.741	.000
Total household expenditure & savings (Income, Y)	1.910E - 03	.000	.134	6.309	.000
How much did you spend on sprays?(MPROTEC)	.976	.212	.109	4.613	.000
How do you classify the economic status of your household relative to others in this community? (SELF)	-513.944	184.388	1.104	-2.787	.005
Total hospital treatment cost (MALCOST)	.339	.098	.078	3.451	.001

a. Dependent variable: willingness to pay for total eradication of malaria

b. Linear regression through the origin

c. Treatment cost in clinic/hospital not involving admission is used

**Other statistics: **R^2 ^= 0.468; DW = 1.709; F = 173.902

**Table 4:** Estimates of the log-linear regression model

	**Unstandardized coefficients**		**Coefficients**^a,b^**Standardized coefficients**		
			
**Model**	**B**	**Std. error**	**Beta**	**T**	**Sig**
**1 **Public Medical facilities	-.125	.069	-.010	-1.822	.069
Log of HH highest level of education	.408	.066	.124	6.203	.000
Log of HH Income	.408	.022	.604	18.466	.000
Log of cost of spraying	.224	.028	.167	7.877	.000
Log self-assessment rating	-.578	.077	-.077	-7.460	.000
Log of total cost of hospital treatment^c^	.210	.032	.167	6.652	.000
Stranger	-.300	.185	-.005	-1.624	.104
Married household	.171	.067	.019	2.552	.011

a. Dependent variable: Log of WTP to eradicate malaria

b. Linear regression through the origin

c. Treatment cost in Clinic/Hospital not involving admission is used

**Other statistics: **R^2 ^= 0.987; DW = 1.522; F = 15341.9

These results indicate that the major determinants of households willingness to pay for malaria eradication and control are their: level of education, income, cost of protection, self assessment of their status relatively to others in the society and total cost of obtaining treatment in the hospital with married households ready to pay more than the singles. Similarly, those currently attending public medical facilities are willing to pay less for malaria control as well as strangers (i.e. those who have not stayed in the community more than one year).

In particular, estimates in Table 4 suggest that a one percentage increase in the number of households' highest years of schooling will bring about a 0.41 percent increase in the amount they are willing to pay for the control of malaria. Similarly, one percentage increase in the household income level will bring about a 0.41 percent increase in the amount they are willing to pay for the control of malaria. Also, a one percentage increase in the cost of spraying room and in the total cost of obtaining treatment in the clinic/hospital would lead to increases of 0.22 and 0.21 percent respectively in the amount they are willing to pay for malaria control.

Furthermore, the results imply the households with married persons, on average, are ready to pay Naira 1245 (USD 10.4) more than the average of Naira 7,324 (USD 61) while those currently using public medical facilities are willing to pay Naira 916 (USD 7.6) less than the group average, with the effect of stranger-status being negative Naira 2,197 {USD 18.3} less than the average) but this effect being not statistically significant.

## Conclusion

The results show a high level of willingness to pay for malaria control in Nigeria. They indicate that if there were insurance policy for malaria treatments, households would be prepared to pay a premium of an average of about Naira 1,112 (USD 9.3) per month to be covered. This is about Naira 427 (USD 3.6) in excess of the average expenditure they currently make on malaria treatment per month. This would be a sort of price for buying certainty.

Similarly, households are willing to pay on the average a sum of Naira 7,324 (USD 61) per month for the control of malaria. Again, this is an excess of about Naira 2,715 (USD 22.6) over the cost they currently bear (protection, treatment and indirect costs), and it represents households' average valuation of their intangible costs of malaria illness. This amount represents about Naira 611.7 (USD 5.1) per head per month and Naira 7,340 (USD 61.2) per year. For a country with a population of about 120 million this translates to about Naira 880,801 million per annum representing about 12.0 per cent of Gross Domestic Product. Hence, the malaria burden in Nigeria is enormous, intolerable and has a devastating impact on economic growth.

We also noted with concern the association between WTP and socio-economic status, and the greater price sensitivity of the lowest economic groups. There is cause for concern about relying on strategies of malaria control that require out-of-pocket contributions from all segments of the population. Access to effective treatment, particularly as Nigeria has recently changed its treatment policy to the more expensive artemisinin based combination therapy, and the selling of nets put to question the prospects of achieving the Abuja targets, and there is an urgent need for strategies to protect the very poor from user fees through carefully designed and targeted subsidies.

In the long term, it is important to recognize that health and poverty are closely linked. Reducing the burden of malaria in Nigeria will help to contribute to the economic well-being of communities; and poverty-reduction will be an essential input into improving health. National malaria control programme in Nigeria and their partners need to recognize these links, and identify mechanisms for ensuring that the poorest have access to essential health interventions.

## Authors' contributions

AJ and TO conceived the study and participated in its design and implementation. AJ did the analysis of the data and participated in the drafting of all sections of the manuscript. OS, AP and TO participated in the drafting of all sections of the manuscript. AJ, OS and AP were involved in data collection. All authors read and approved the final manuscript.

## Supplementary Material

Additional File 1 Willingness to pay questions employed. Details of questions used to determine willingness to pay Click here for file

Additional File 2 Variables and the measurement. Detailed explanation of variables and their measurement Click here for file

Additional File 3 Household expenditure. This format was used to collect information on household expenditure Click here for file
