# Supplementary material for: Quantifying the economic burden of malaria in Nigeria using the willingness to pay approach
Source: Cost Eff Resour Alloc. 2007 May 22;5:6. doi: 10.1186/1478-7547-5-6 (PMC1890276; doi:10.1186/1478-7547-5-6)
Supplement: Additional File 1 — Willingness to pay questions employed. Details of questions used to determine willingness to pay [file 1478-7547-5-6-S1.doc]

Additional file 1: willingness to pay questions employed

***Explanatory notes****: Malaria is a parasitic disease transmitted by Anopheline mosquitoes from an infected human to other human beings when they bite humans*. *In order to ensure that respondents have a common understanding of malaria, the following symptoms would be taken as indicative of malaria:*

*For children: Hot body; Fever; weakness; sleepiness; loss of appetite; vomiting.*

*For adults: Headaches; weakness and fever; aches and pain; temperature; bitterness of mouth.*

*Interviewers are, therefore, requested to let respondents have a clear understanding of what malaria is and this is best ascertained by asking them relevant questions about its symptoms and if need be educate them.*

Information, product features and questions

Malaria disease is very serious in Nigeria. It is the number one cause of morbidity, accounting for 40 – 60% of all outpatient hospital attendance in the country.

Apart from this, it is also the leading cause of mortality in children under 5 years. It is also the leading cause of workdays lost due to illness.

The effect of malaria on people of all ages is quite disturbing. It is however very serious among pregnant women and children. When malaria infection is not properly treated in pregnant women, it can cause anaemia and also lead to miscarriages, underweight babies and maternal deaths.

In children, frequent severe malaria attacks not only adversely affect their attendance at school but may also affect their brains, which can retard their learning abilities at school.

In addition to time and money spent on fighting malaria, it causes considerable pain and weakness among its victims. This can reduce peoples working abilities. All these have negative effects on household incomes and national development.

It is therefore necessary that households protect themselves against malaria and also seek prompt but effective treatment anytime malaria occurs in the household. It may cost between N200 and N1,200 to treat one episode of malaria attack.

With this in mind and having experienced the pains and suffering associated with malaria illness by yourself and members of your household before, I would be very grateful if the following questions are adequately answered for me. Thank you.

Features of medical care

Some non-governmental organizations are planning to work out a programme with government and private health care providers. Under the arrangement, the health care facility where you currently seek medical help (hospital/clinic/ primary health care centre) will be adequately staffed, provided with modern facilities (including laboratories) and well stocked with all drugs needed to effectively treat malaria (i.e. ensure that all symptoms abate within the first day of treatment and it is completely cured after fifth day of treatment). But because this programme is not funded by government it has to be financed from payments made by households that would be attending the medical centres and without payment by patients the programme will not be run. Under the programme, every household would be required to pay an agreed amount per month per adult member of the household and an agreed amount per month per child member of the household to be covered. Payment shall be at cash office of the medical centre and receipt shall be issued for every payment. A covered person would just have to walk into the medical centre when suffering from malaria with proper identification to receive effective malaria treatment.

1. Would this household be willing to pay for effective malaria treatment (Medical care) when a member contracts the disease at this price per month? [**Stop going further if respondent answers in the affirmative and ask for the exact amount that he is willing to pay]**

**a. Adult ?**

| **Value (Naira)** | **Yes = 1; No = 2** |
| --- | --- |
| **2000** |  |
| **1000** |  |
| **800** |  |
| **500** |  |
| **400** |  |
| **300** |  |
| **200** |  |
| **100** |  |

What is the maximum amount that you are willing to pay per month per adult for effective treatment of malaria?

**b. Child?**

| **Value (Naira)** | **YES = 11; NO = 2** |
| --- | --- |
| **2000** |  |
| **1000** |  |
| **800** |  |
| **500** |  |
| **400** |  |
| **300** |  |
| **200** |  |
| **100** |  |

What is the maximum amount that you are willing to pay per month per child for effective treatment of malaria?

**Features of insecticide-treated bed net**

Insecticide-treated nets (ITNs) are nets treated with special chemicals. The ITNs repel and kill mosquitoes, and also ensure that mosquitoes do not get access to persons covered by it and it is significantly more effective than ordinary mosquito nets. These treated nets reduce significantly the number of times one gets knocked down by malaria say from 4 times a year to about 2 a year. The net is to be re-treated yearly at about a third of its original. An NGO is planning a programme that would distribute these nets to households on a cash-and-carry basis. The beauty of the arrangement is that the nets will always be available when you need them and re-treatment opportunity will always be available. The nets are in two sizes, namely, small and family-bed size.

2. How much would this household be willing to pay to avoid malaria attack (protection) by buying:

1. **Bed nets** (ITN)

| **Value (Naira)** | **Yes = 1 No = 2** |
| --- | --- |
| 3 000 |  |
| 2 500 |  |
| 2 000 |  |
| 1 500 |  |
| 1 000 |  |
| 750 |  |
| 500 |  |
| 250 |  |

(2a.ii.) What is the maximum amount that you are willing to pay to be protected by nets (ITN)?

**Features of area spraying**

Area spraying involves spraying the entire community with special chemicals that kill mosquitoes and their eggs/lava. This is done by designated officials and is normally done twice a year and is known to significantly reduce the number of times one gets knocked down by malaria say from 4 times a year to about 5 in 2 years.

2b. How much would this household be willing to pay to avoid malaria attack (protection) by supporting:

Area spraying [twice a year]

| **Value (Naira)** | **Yes = 1 No = 2** |
| --- | --- |
| 3 000 |  |
| 2 000 |  |
| 1 000 |  |
| 750 |  |
| 500 |  |
| 200 |  |

What is the maximum amount that you are willing to pay per year for area spraying?

**Features of malaria total eradication programme**

The programme will be a combination of area spraying (twice a year) and routine check up of those covered to discover and treat people with malaria parasites in the blood samples before malaria illness develops. If a malaria vaccine is found in future, it will also include the administration of the vaccine to ensure that the probability of one suffering from malaria will be less than one per cent and in such cases victims will be treated promptly and free of charge.

3. Considering (Q1-2) and noting that your current expenses on malaria treatment and prevention is about …………: How much would this household be willing to pay (in total) per annum to eradicate malaria attack on this household?(***Interviewers are expected to quickly add up the relevant current costs from respondent’s answers to earlier questions on cost of illness***)

| **Value (Naira)** | **Yes = 1 No = 2** |
| --- | --- |
| 50 000 |  |
| 25 000 |  |
| 20 000 |  |
| 15 000 |  |
| 10 000 |  |
| 7 500 |  |
| 5 000 |  |
| 3 000 |  |
| 2 500 |  |
| 2 000 |  |
| 1 000 |  |
| 750 |  |
| 500 |  |

(3b.) What is the maximum amount that you are willing to pay per year for total eradication of malaria?
