# Supplementary material for: Quantifying the economic burden of malaria in Nigeria using the willingness to pay approach
Source: Cost Eff Resour Alloc. 2007 May 22;5:6. doi: 10.1186/1478-7547-5-6 (PMC1890276; doi:10.1186/1478-7547-5-6)
Supplement: Additional File 2 — Variables and the measurement. Detailed explanation of variables and their measurement [file 1478-7547-5-6-S2.doc]

Additional file 2: Variables and the measurement

| Variable short name | Description | Variable type |
| --- | --- | --- |
| WTPMC | Willingness to pay for malaria eradication as indicated by each respondent; | Real number |
| Y | Household’s income (Sum of expenditures & savings) | Real number |
| EDUC | Years of schooling of household’s head | Real number |
| MPROTEC | Household’s current malaria protection expenditures | Real number |
| MALCOST | Household’s current malaria treatment expenditures | Real number |
| INDIRECT COST | Value of household’s lost output and time due to malaria illnesses | Real number |
| SELF | Household’s self-assessment – a measure of wealth and relative income; value varies from 1 -5 | Categorical |
| MARRIED | Household’s marital status: Married = 1; Others = 0 | Categorical |
| STRANGER | Household’s length of stay in the community: Less than 1 year = 1; Others = 0 | Categorical |
| PUBMED | Household Using public facilities for malaria Treatment: Those currently Using public facilities = 1; Others = 0 | Categorical |
