# Supplementary material for: Quantifying the economic burden of malaria in Nigeria using the willingness to pay approach
Source: Cost Eff Resour Alloc. 2007 May 22;5:6. doi: 10.1186/1478-7547-5-6 (PMC1890276; doi:10.1186/1478-7547-5-6)
Supplement: Additional File 3 — Household expenditure. This format was used to collect information on household expenditure [file 1478-7547-5-6-S3.doc]

Additional file 3: Household expenditure

List the expenditures your household members have incurred either within the last one day or last one week or last one month or last one year (whichever is appropriate) (Naira)

[Per day/ per week/per month/ per year] ***(Circle the appropriate)***

| Items of expenditure & savings | Amount (Naira) |
| --- | --- |
| a. Food |  |
| 1. Market |  |
| ii. Own produce (Inputted cost) |  |
| b. Clothing |  |
| c. Toiletries (soap, etc) |  |
| d. House rent |  |
| e. Housing utilities (water, light, telephone) |  |
| f. Fuel [charcoal, firewood, gas etc] |  |
| g. Health care |  |
| h. Transportation |  |
| i. Education (books, fees, etc.) |  |
| j. Donation, gifts & remittances |  |
| k. Farm inputs (other than labour) |  |
| l. Farm machinery (rental) |  |
| m. Farm labour |  |
| n. Raw materials (Artesian producer) |  |
| o. Savings |  |
| p. Levies [church, project, funerals] |  |
| q. Household durables [car, TV, etc] |  |
| r. Immovable assets [e.g. land] |  |
| s. Others (specify) … |  |
